# The Complexity of Transferring Remote Monitoring and Virtual Care Technology Between Countries: Lessons From an International Workshop

**DOI:** 10.2196/46873

**Published:** 2023-08-01

**Authors:** Quynh Pham, David Wong, Kaylen J Pfisterer, Dionne Aleman, Nick Bansback, Joseph A Cafazzo, Alexander J Casson, Brian Chan, William Dixon, Gerasimos Kakaroumpas, Claudia Lindner, Niels Peek, Henry WW Potts, Barbara Ribeiro, Emily Seto, Charlotte Stockton-Powdrell, Alexander Thompson, Sabine van der Veer

**Affiliations:** 1 Centre for Digital Therapeutics University Health Network Toronto, ON Canada; 2 Institute for Health Policy, Management and Evaluation Dalla Lana School of Public Health University of Toronto Toronto, ON Canada; 3 Tefler School of Management University of Ottawa Ottawa, ON Canada; 4 Department of Computer Science The University of Manchester Manchester United Kingdom; 5 Division of Informatics, Imaging and Data Sciences Faculty of Biology, Medicine and Health, Manchester Academic Health Science Centre The University of Manchester Manchester United Kingdom; 6 Department of Systems Design Engineering University of Waterloo Waterloo, ON Canada; 7 Department of Mechanical & Industrial Engineering University of Toronto Toronto, ON Canada; 8 School of Population and Public Health University of British Columbia Vancouver, BC Canada; 9 Department of Electrical and Electronic Engineering The University of Manchester Manchester United Kingdom; 10 EPSRC Henry Royce Institute Manchester United Kingdom; 11 KITE Research Institute Toronto Rehabilitation Institute University Health Network Toronto, ON Canada; 12 Centre for Epidemiology Versus Arthritis University of Manchester Manchester United Kingdom; 13 Alliance Manchester Business School The University of Manchester Manchester United Kingdom; 14 Institute of Health Informatics University College London London United Kingdom; 15 Manchester Institute of Innovation Research Alliance Manchester Business School The University of Manchester Manchester United Kingdom; 16 Manchester Centre for Health Economics, Division of Population Health Faculty of Biology, Medicine and Health, Manchester Academic Health Science Centre The University of Manchester Manchester United Kingdom

**Keywords:** digital health innovation, digital health intervention, digital health landscape, digital health solution, health care system, regulatory pathway, remote monitoring, remote monitoring, technology transfer, virtual care

## Abstract

International deployment of remote monitoring and virtual care (RMVC) technologies would efficiently harness their positive impact on outcomes. Since Canada and the United Kingdom have similar populations, health care systems, and digital health landscapes, transferring digital health innovations between them should be relatively straightforward. Yet examples of successful attempts are scarce. In a workshop, we identified 6 differences that may complicate RMVC transfer between Canada and the United Kingdom and provided recommendations for addressing them. These key differences include (1) minority groups, (2) physical geography, (3) clinical pathways, (4) value propositions, (5) governmental priorities and support for digital innovation, and (6) regulatory pathways. We detail 4 broad recommendations to plan for sustainability, including the need to formally consider how highlighted country-specific recommendations may impact RMVC and contingency planning to overcome challenges; the need to map which pathways are available as an innovator to support cross-country transfer; the need to report on and apply learnings from regulatory barriers and facilitators so that everyone may benefit; and the need to explore existing guidance to successfully transfer digital health solutions while developing further guidance (eg, extending the nonadoption, abandonment, scale-up, spread, sustainability framework for cross-country transfer). Finally, we present an ecosystem readiness checklist. Considering these recommendations will contribute to successful international deployment and an increased positive impact of RMVC technologies. Future directions should consider characterizing additional complexities associated with global transfer.

## Background

Remote monitoring and virtual care (RMVC) technologies are an increasingly common class of digital health innovation, estimated to be worth US $143 billion in 2023 [1]. RMVC technology enables patients to electronically collect health data outside of traditional care settings using internet-connected devices (eg, smartphones and sensors) and transmit these data to share it with health care professionals. This has the potential to increase access to and productivity of services and improve patient outcomes [2,3]. One way to efficiently increase the positive impact of RMVC technologies is to transfer successful products between countries. Given the similarities between Canada and the United Kingdom, one may expect the transfer of digital health innovations between the 2 countries to be relatively straightforward. Similarities pertain to payment structure and health insurance coverage, some population demographics (proportion urban, aging, etc), supports to facilitate data sharing and data-driven insights (electronic health records, data science initiatives, etc), and the presence of academic teaching hospitals (Table 1).

Although there have been successful examples, including the Maple virtual care platform and Ieso digital cognitive behavioral therapy [4,5], these have been scarce and accompanied by similar high-profile instances in which the rollout of RMVC technologies was slower than anticipated [6]. We, therefore, aimed to increase our understanding of factors that may influence the successful transfer of RMVC technology between countries, using the transfer between Canada and the United Kingdom as an example.

**Table 1:** Examples of similarities between Canada and the United Kingdom as contexts for deploying remote monitoring and virtual care (RMVC) technology.

Area of similarity	Canada	United Kingdom
Health care system	Centralized and publicly funded by province or territory (eg, Ontario Health Insurance Plan)	Centralized and publicly funded by devolved nation (eg, National Health Service England)
Urban population	82% of people live in urban areas	84% of people live in urban areas
Health challenges and outcomes	High prevalence of long-term conditions; life expectancy of 82 years	High prevalence of long-term conditions; life expectancy of 81 years
Burgeoning digital health sectors, including data science and artificial intelligence	Vector Institute; Digital Technology Supercluster; Toronto Innovation Acceleration Partners	Alan Turing Institute; Health Data Research UK; Health Innovation Manchester
Robust administrative and electronic health record research databases	Institute for Clinical Evaluative Sciences in Ontario, PopData BC	Clinical Practice Research Datalink
Academic and hospital networks	Academic health science networks (eg, Health Research BC)	Academic health science centers (eg, Manchester Academic Health Science Centre)
Paying out-of-pocket and health insurance coverage	Some out-of-pocket and health insurance payment for certain health care services (eg, dental and medications)	Some out-of-pocket payment for certain health care services in specific populations (eg, dental [age >18 years] and medication [ages 16-60 years])

## International Workshop With Experts

### Overview

Our objective was to identify factors that may add complexity when transferring RMVC technology between Canada and the United Kingdom. We organized a workshop with 15 experts from both countries on behalf of the International Centre for Translational Digital Health [7]. Initially, when this workshop was held, this center was a collaboration between the University of Toronto (Canada) and the University of Manchester (United Kingdom); it now includes the University of Melbourne (Australia) since 2022 [7]. Workshop participants were experts in medical engineering, health informatics, clinical medicine, and health policy and implementation. They examined 3 cases representing different RMVC technologies (Textboxes 1-3), with group discussions focusing on identifying differences between Canada and the United Kingdom that might add complexity when transferring the example RMVC from one country to the other.

**Example RMVC technology 1: neural network–assisted sampling for intermittent ambulatory blood pressure measurements.**

**Figure 1:**
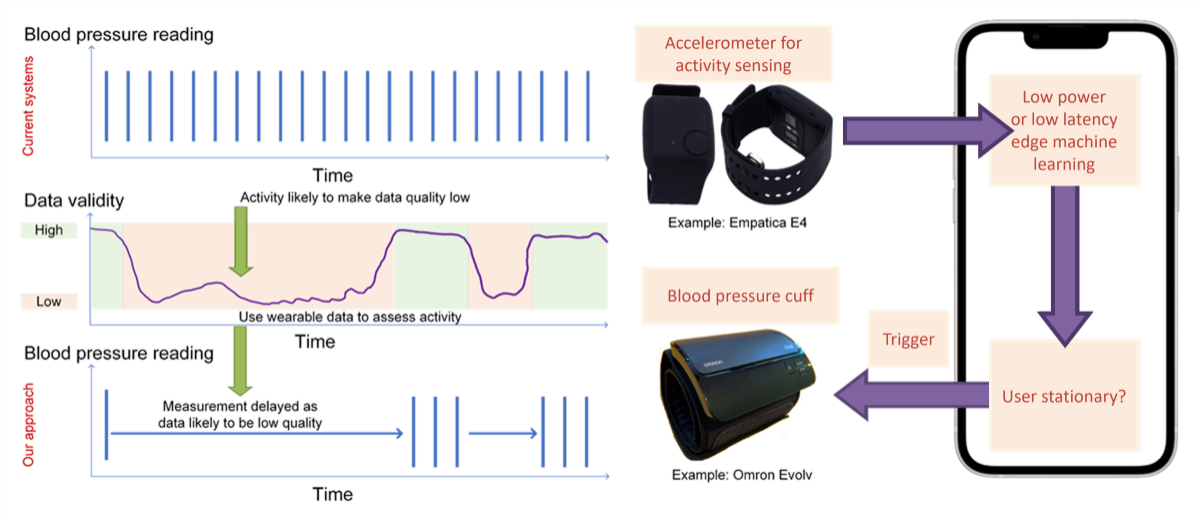
Neural network sampling for intermittent ambulatory blood pressure measurements.

The aim of the system (Figure 1), developed at the University of Manchester, is to reduce the burden of taking ambulatory blood pressure readings by requiring fewer readings to still obtain the same underlying average. This is intended to reduce burden on users as cuffs will inflate less often (which is uncomfortable) and to reduce burden on caregivers or clinicians who will have fewer readings to interpret.

Blood pressure varies with activity, and guidelines for in-clinic blood pressure monitoring ask participants to sit stationary for 5 minutes before a reading. Home-based, ambulatory-based, and wearable-based blood pressure monitoring are widely available, but they do not account for this. The system uses human activity recognition to allow the “5 minute” stationary condition to be applied to out-of-the-clinic measurements. The novel device requires users to wear an accelerometer device for monitoring motion, and this is used to determine whether they have met the measurement requirements before a reading is taken. It is an example of using machine learning for data-driven adaptive sampling. A complete prototype exists and is ready for preclinical testing.

**Example RMVC technology 2: Remote Monitoring of Rheumatoid Arthritis (REMORA), smartphone-based symptom tracking for rheumatoid arthritis integrated into electronic health records systems.**

**Figure 2:**
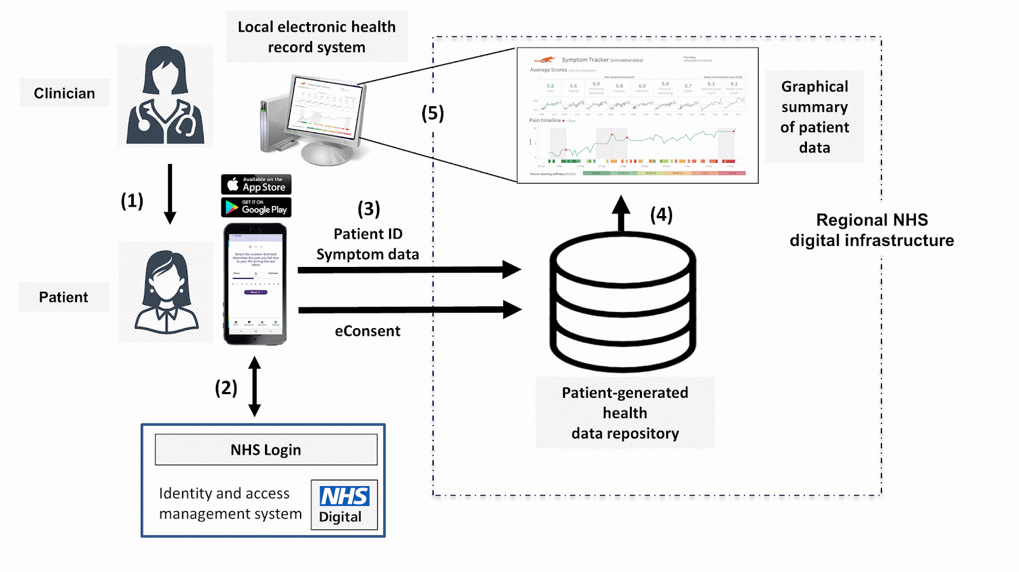
Remote Monitoring of Rheumatoid Arthritis, a smartphone-based symptom tracking for rheumatoid arthritis integrated into electronic health records systems.

REMORA is a United Kingdom research program to develop, evaluate, and scale up a complex intervention that enables people with rheumatoid arthritis to daily track their symptoms on their smartphone to improve clinical care, self-management, and outcomes (Figure 2).

The diagram above shows that clinicians prescribe remote symptom tracking to patients (1), who then download the REMORA app from the app store. After authenticating themselves using the login functionality of the National Health Service (NHS) (2), they start tracking their symptoms. Symptom data is sent to a data repository within a secure, regional NHS environment, alongside the patient’s e-consent for secondary use of their symptom data for research (3). A visualization summarizes the symptom data graphically (4), which clinicians can access within their local electronic health record to discuss with patients as part of the consultation (5).

A successful proof-of-concept study in 2015 integrated and displayed daily symptom tracking data from the app in the electronic health record at a single hospital site. It showed that patients and clinicians considered the system feasible and beneficial [8], as well as how shared insights from the symptom data may change the power dynamics of the consultation [9]. In 2021, we established and tested a scalable infrastructure to deploy our proof-of-concept, which enabled its evaluation in an ongoing multisite trial in all rheumatology outpatient departments across Greater Manchester and North West London.

**Example RMVC technology 3: Medly, a smartphone–based heart failure management program.**

**Figure 3:**
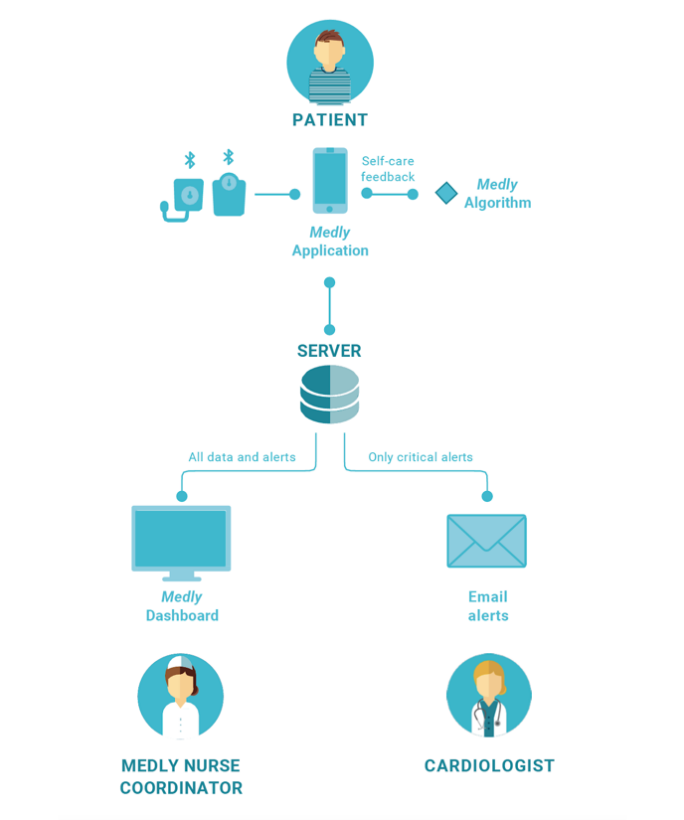
Medly, a smartphone-based heart failure management program.

Medly was developed at the University Health Network in Toronto, Canada. Medly consists of two core components (Figure 3): (1) the *Medly system* to support active monitoring of heart failure patients, clinical management, and patient self-care; (2) *Medly Service* that includes key people (ie, clinicians providing the clinical services) and the processes required to operationalize the Medly System.

The *Medly app* prompts patients to enter their daily readings, including weight, blood pressure, heart rate, and heart failure–related symptoms. Patients use a Bluetooth weight scale and blood pressure monitor to send readings automatically or manually enter the values on the app. The *Medly algorithm*, a rules-based expert system validated by heart failure specialists, immediately analyzes the entered readings against set personalized thresholds and provides the patient with instant feedback and instructions. Automated alerts are also sent to the patient’s care team (usually a nurse) when readings are outside of their predefined thresholds for further assessment and triage.

The *Medly dashboard* provides Medly clinicians with real-time contextualized data on their patients’ clinical status and recent symptoms consistent with acute exacerbations. Clinicians also use secure email and messaging to review and respond to patient alerts.

Medly was developed following ISO 13485 Medical Device Quality Management standards and is rated by Health Canada as a Class II Software as a Medical Device. It is used as standard of care at the University Health Network since 2016 and has been deployed as standard of care at some other health care institutions across Ontario. A body of evidence on its clinical effectiveness and implementation outcomes exists through several trials and evaluations [10-12].

### Guiding Framework

We grounded our workshop discussion on the complexity of adoption by using 3 example technologies. Each technology was assessed using the evidence-based and theory-informed nonadoption, abandonment, scale-up, spread, sustainability (NASSS) framework [13]. The NASSS framework is used to both predict and evaluate the complexity of technology in health care (or social care) [13]. It comprises 7 dimensions to rate the complexity of the technology: condition, technology, value proposition, adopters, organization, wider system or external context, and embedding and adaptation over time. The workshop discussion focused on all NASSS dimensions except embedding and adaptation over time (pertaining to later-stage technologies with refined deployment over time), in addition to questions from the short NASSS complexity assessment tool [14]. More specifically, each technology was evaluated for (1) its assessment of complexity while considering application between different jurisdictions, (2) the importance placed on the complexity as a barrier to implementation success, and (3) the degree to which it would be the same or different between jurisdictions. After the workshop, we analyzed contemporaneous notes and audio recordings of the discussions to extract key themes from the complexities, described next.

## Complexities for Transferring RMVC Technology Between Canada and the United Kingdom

There were several apparent similarities between Canada and the United Kingdom that, upon further probing, yielded nuanced differences where unexpected complexity could arise; we describe these below. They are intended as key illustrative examples rather than an exhaustive list.

### Differences in Addressing Needs of Minority Groups

The Indigenous peoples of Canada comprise 5% of the total population in Canada; this significant minority group is not present in the United Kingdom [15]. This adds an important layer of culturally compassionate considerations for equitable access to RMVC technology transfer. Both Canada and the United Kingdom have significant immigrant populations. By 2031, 32% of Canada’s population and 15% of the United Kingdom’s population will comprise ethnic minorities [16,17]. The COVID-19 pandemic has escalated RMVC from relative novelty to necessity for timely access to services in both countries. However, minority communities, who may have worse access to technology, poorer capacity to use technology due to lower digital literacy, and inferior outcomes related to technology use [18-20], were unable to benefit equally from this shift in service delivery. From discussing REMORA (Textbox 2) and Medly (Textbox 3), it appeared that efforts to address such ethnic health inequalities in the United Kingdom did not focus on any one minority group. However, in Canada these focused primarily on the Indigenous population, requiring additional considerations to ensure RMVC solutions are created in authentic partnership. More broadly, special jurisdictions (ie, Quebec, Scotland, Northern Ireland, and Wales) may add additional complexity for implementation at the National level. Without accounting deeply for these needs from the outset, vendors may experience unanticipated cultural and governmental barriers to transfer (eg, culturally appropriate adaptations, language differences: Canadian French vs Canadian English vs France French).

### Differences in Physical Geography

Canada’s physical geography is substantially more expansive than that of the United Kingdom, implying much larger climate variation and greater distances to clinical centers providing a differential incentive for RMVC. Since temperature can diminish the reliability of remote monitoring devices [21], differences in climate between the United Kingdom and parts of Canada were identified as a potential complexity when discussing the ambulatory blood pressure measurement system (case 1; see Textbox 1). Canada’s geography also impacts communication infrastructure, including the price and reliability of high-speed internet. For example, 92% of rural areas in the United Kingdom have access to superfast, relatively cheap broadband [22] versus less than 50% of rural Canada [23]. This means that Canadians, particularly those in more rural areas, may encounter higher barriers to accessing reliable RMVC than rural populations of the United Kingdom. We identified this as a key complicating factor for transferring REMORA (case 2) from the United Kingdom to Canada.

### Differences in Clinical Pathways

Implementing new RMVC technology almost always involves some integration into existing clinical pathways. Alignment between the technology on the one hand and the pathways and associated organizational routines on the other will, therefore, affect the technology’s acceptance and scalability. When discussing cases 1 and 2, we considered differences in clinical pathways to have a limited impact on complexity. This was mainly because these technologies, in their current form, focus on providing more complete and accurate information to inform clinical decisions within existing pathways. So, once they are technically integrated into electronic health record systems, they require relatively minor changes to their ways of working. However, Medly (case 3) involved a change from consultant-led to nurse-led delivery of heart failure services. Interestingly, for transfer from Canada to the United Kingdom, this would not necessarily add complexity because nurse-led care models are more common in the United Kingdom compared to Canada for a range of conditions, including heart failure [24]. However, it would complicate transfer of a similar RMVC technology in the other direction.

### Differences in Value Propositions

Creating a cohesive set of value propositions that meets the demands of patients [25] as well as other stakeholders (eg, policy makers, regulators, and payers) can be challenging [26]. This is partly because countries have only recently developed frameworks that define how value should be conceptualized for RMVC and other digital health technologies, and what evidence is required to demonstrate this value. For example, in 2018, the National Institute for Health and Care Excellence (NICE) for England and Wales developed the evidence standards framework for digital health technologies [27], while Canada only recently launched the Canadian Network for Digital Health Evaluation in collaboration with pan-Canadian organizations to start developing such frameworks [28]. Companies can increase the value of their product by incorporating NICE guidelines [27,29], meeting the Digital Technology Assessment Criteria for health and social care [30,31], and taking their technology through the Innovation and Technology Payment program [32].

The challenge of developing value propositions is amplified when transferring technologies between countries. Even between countries with publicly funded health care systems, there may be substantial variation in how services are funded. In the United Kingdom, for example, innovations such as RMVC are typically commissioned by regional policy makers in Integrated Care Systems [33], whereas in Canada, commissioning RMVC technology usually happens at the local practice level. Another example is that, in Canada, clinicians are generally paid according to the procedures used to treat a patient (fee-for-service), while in the United Kingdom, primary care clinicians are mainly paid through a set amount per patient (capitation). This implies that aiming to, for example, reduce the duration of visits, such as for Medly (case 3), may be attractive for uptake in the United Kingdom, but may face barriers to adoption in Canada if that leads to decreased income for clinicians.

### Differences in Government Priorities and Support for Digital Innovation

Despite the mandate and priority identified by Canadian Federal and Provincial bodies to innovate virtual care delivery during the pandemic (including CAD $240.5 [US $180.5] million investment [34]), the Ontario Medical Officer Health released a letter urging physicians to avoid providing virtual care in lieu of in-person care [35]. The 2022 Canadian virtual care policy framework update continues to scope RMVC technologies solely as “an additional channel for access that complements traditional face-to-face models of care” [34]. In contrast, the United Kingdom directly incorporates digital health into policy documents such as the NHS Long Term Plan [36], Digital First Primary Care [37], and the 2022 Plan for Digital Health and Social Care [38]. The United Kingdom has also increased investment in digital innovation directly within the NHS as well as through academic and industry routes. For example, the NHS Artificial Intelligence Laboratory recently funded £140 (US $177.61) million worth of AI Health and Care Awards [39]. RMVC will likely remain a high priority in the United Kingdom, given that both the current government [40] and the main opposition party [41] have assigned an important role to digital transformation in solving NHS pressures. This difference in government priorities and support arose as a key complexity for cases 1 and 2, where there would be transfer from the United Kingdom to Canada.

### Differences in Regulatory Considerations

Regulations for digital technologies in various sectors, including health care, are continually evolving in both countries. And while the general flavor of technology regulations is similar between Canada and the United Kingdom, there are some critical differences. In Canada, RMVC technologies that are considered medical devices are licensed through Health Canada and regulated at the level of individual clinicians by the Colleges of Physicians and Surgeons of its different provinces or territories. In the United Kingdom, regulation is the responsibility of government bodies (eg, Care Quality Commission in England) or independent regulators (eg, General Medical Council, Medicines and Healthcare Products Regulatory Agency, and notified bodies for applying for CE (conformité européenne) mark and United Kingdom Conformity Assessed marking). In the context of case 3, we discussed how this would increase complexity when transferring Medly to the United Kingdom because Canadian innovators need to be aware of and comply with the most up-to-date regulations of the United Kingdom. However, they could collaborate with the United Kingdom’s partners early on to start preparing an application for CE marking and the Innovation and Technology Payment program by submitting the available evidence for Medly’s clinical and cost-effectiveness, developing a scalable growth plan, and assessing whether they could rapidly meet an increasing demand for their product once approved. It is worth noting that most regulation for remote and virtual care delivery overlaps with face-to-face forms [42]. This means that, in addition to having to navigate the requirements specific for RMVC technology, innovators transferring their technologies from one country to the other need to engage with the broader regulatory landscape for health care delivery.

Two additional considerations for legislation and policy should include an assessment of approaches to procurement and approaches to privacy and security, which are both nuanced but can significantly impact transferability. For example, some jurisdictions require public sector procurement at the local, regional, or national levels. In Canada, it may be more straightforward to make direct partnerships (eg, directly contacting clinical champions). Within the United Kingdom, for optimized success of uptake, taking a top-down (eg, regional- or national-level support) and bottom-up (local-level support) approach is needed for movement. Similarly, differences in data-privacy regulations may affect the ability to transfer technology. For example, the General Data Protection Regulation (GDPR) [43] act provides strict data privacy guidelines in Europe around data storage and use compared to Canada’s Personal Information Protection and Electronic Documents Act (PIPEDA) [44,45]. This may make the spread easier in countries with stricter data privacy guidelines, but more difficult in the reverse direction. Differences in data-privacy guidelines may also impact the vendor’s business case (ie, prohibitive vs profitable use of data and data residency).

## Discussion and Recommendations

We identified several key differences between 2 countries that, at first glance, seem very similar. This suggests that one should anticipate significant complexity and barriers when considering the international deployment of RMVC technologies. We found that the anticipated increase in complexity often had a particular direction. For example, more detailed frameworks for regulations and value propositions in the United Kingdom hamper transfer from Canada, but not vice versa. Based on these differences and their directionality, we provide an overview of lessons learned (Table 2) with an initial “checklist” of key considerations for transferring RMVC technology from one country to the other (Table 3).

**Table 2:** Summary of key differences, lessons learned, and anticipated directionality for transferring remote monitoring and virtual care (RMVC) technology between Canada and the United Kingdom.

Key difference	Directionality	Workshop learnings and recommendations
Addressing unmet needs of minority groups	Increases complexity when transferring RMVC technology from the United Kingdom to Canada	For countries with deeply rooted colonization histories and Indigenous communities, such as Canada, it is more complex to understand equity and undertake meaningful cocreation.
Physical geography	Increases complexity when transferring RMVC technology from the United Kingdom to Canada	Climate differences or geographical range can affect RMVC operationalization, from temperature sensitivity in inclement weather conditions to service availability in remote areas. “Remote” does not mean the same in Canada compared to the United Kingdom.
Clinical pathways	Contingent on degree of alignment of pathways	Alignment of the RMVC technology with existing clinical pathways will affect its acceptance and scalability. For example, it may be easier to transfer nurse-led models of care to the United Kingdom where this is more common compared to in Canada.
Value propositions	Contingent on (available evidence for) cost-effectiveness of RMVC technology	Defined costing structures and evidence frameworks affect uptake. For example, transfer from capitation-based funding in the United Kingdom to fee-for-service in Canada may add complexity; comparable evidence for efficacy acceptable in the United Kingdom may be insufficient for adoption in Canada.
Government priorities and support for digital innovation	Increases complexity when transferring RMVC technology from the United Kingdom to Canada	Incorporation of digital health and RMVC technology into health care strategies and policies in the United Kingdom is currently more mature than in Canada. This may change with time.
Regulatory considerations	Increases complexity when transferring RMVC technology from Canada to the United Kingdom	Regulatory pathways in the United Kingdom consist of several elements without offering a clear and single point of access. This makes them harder to navigate compared to in Canada, where Health Canada licenses RMVC technology.

**Table 3:** Recommendations for transferring remote monitoring and virtual care (RMVC) technology between countries with similar health care systems (ie, Canada and the United Kingdom).

	Recommendation
Addressing unmet needs of minority groups	Assess appropriateness of RMVC technology to provide compassionate and culturally sensitive care. This includes cocreation with partners and ensuring interventions are Indigenous-directed where appropriate.
Physical geography	Characterize physical RMVC system requirements to ensure compatibility with the intended deployment context. Establish a shared understanding of the definition of “remote” in each country early on.
Clinical pathways	Analyze and map existing clinical pathways and assess the degree of change required for implementing RMVC technology in the intended deployment context.
Value propositions	Ensure an understanding of the costing structure and required evidence base to inform perceived value of the RMVC technology in the intended deployment context.
Government priorities and support for digital innovation	Understand governmental priorities to assess feasibility, inform points of synergy, and explore opportunities for acceleration.
Regulatory considerations	Map regulatory pathways between countries to identify key alignments and divergences.
Supplementary recommendations	Formally consider whether the country-specific differences we highlighted may impact your RMVC technology, and identify ways to overcome the challenges related to these differences Identify which pathways are available to you as an innovator to support cross-country transfer. Report on and apply learnings from the regulatory barriers and facilitators identified in previous attempts to transfer and deploy digital health technologies. Explore what guidance exists on how to transfer digital health technologies across countries, and identify what further guidance may need developing (eg, an extension of the NASSS^a^ framework [14] for cross-country transfer).

^a^NASSS: nonadoption, abandonment, scale-up, spread, sustainability.

As part of future work around facilitating international deployment of RMVC technology, conducting similar workshops could explore differences between countries with comparable health care systems (eg, Australia and the Netherlands). Additionally, nuances of complexities in scaling, spreading, and sustaining technologies between countries with more dissimilar infrastructure, like the health care system, warrant further exploration. More dissimilar health care systems will impart additional complexities across NASSS domains, such as socio-cultural influences of condition management (eg, family shared care), potentially increased disparities in health and digital literacies and technology access, value proposition (who will benefit most), extent of changes needed to routines, how adoption funding decisions are made, as well as additional regulatory impacts, all of which will ultimately affect the embedding and adaptation of technology over time. More broadly, we advocate the creation of a space to publish on successful and failed cross-country transfers of digital health and RMVC technology. This will encourage and enable the digital health research community to share their experiences and findings more widely, thereby expediting learning about the technology transfer and deployment process. Given the reality of the global digital divide, such learning is pertinent for improving equitable access to remote and virtual care services. While outside the scope of this viewpoint paper, as evidence is generated, future work to derive an evidence-based model such as an updated NASSS targeting transfer between countries would be invaluable for directing and prioritizing international and global technology transfer efforts.

## Abbreviations

CE: conformité européenne
NASSS: nonadoption, abandonment, scale-up, spread, sustainability
NICE: National Institute for Health and Care Excellence
RMVC: remote monitoring and virtual care

## References

[1] (2021). Telehealth market size, share and COVID-19 impact analysis, by type (products and services), by application (telemedicine, patient monitoring, continuous medical education, and others), by modality (real-time (synchronous), store-and-forward (asynchronous), and remote patient monitoring), by end-user (hospital facilities, homecare, and others), and regional forecast, 2023-2030. Fortune Business Insights.

[2] Noah B, Keller MS, Mosadeghi S, Stein L, Johl S, Delshad S, Tashjian VC, Lew D, Kwan JT, Jusufagic A, Spiegel BMR (2018). Impact of remote patient monitoring on clinical outcomes: an updated meta-analysis of randomized controlled trials. NPJ Digit Med.

[3] Snoswell CL, Taylor ML, Comans TA, Smith AC, Gray LC, Caffery LJ (2020). Determining if telehealth can reduce health system costs: scoping review. J Med Internet Res.

[4] (2020). Maple.

[5] Online therapy. ieso.

[6] Labine J (2020). AHS pauses partnership with Big White Wall following complaints about name. Edmonton Journal.

[7] (2022). International Centre for Translational Digital Health.

[8] Austin L, Sharp CA, van der Veer SN, Machin M, Humphreys J, Mellor P, McCarthy J, Ainsworth J, Sanders C, Dixon WG (2020). Providing 'the bigger picture': benefits and feasibility of integrating remote monitoring from smartphones into the electronic health record: Findings from the Remote Monitoring of Rheumatoid Arthritis (REMORA) study. Rheumatology (Oxford).

[9] Laverty L, Gandrup J, Sharp CA, Ercia A, Sanders C, Dowding D, Dixon WG, van der Veer SN (2022). Using patient-generated health data in clinical practice: how timing influences its function in rheumatology outpatient consultations. Patient Educ Couns.

[10] Ware P, Ross HJ, Cafazzo JA, Boodoo C, Munnery M, Seto E (2020). Outcomes of a heart failure telemonitoring program implemented as the standard of care in an outpatient heart function clinic: pretest-posttest pragmatic study. J Med Internet Res.

[11] Seto E, Leonard KJ, Cafazzo JA, Barnsley J, Masino C, Ross HJ (2012). Mobile phone-based telemonitoring for heart failure management: a randomized controlled trial. J Med Internet Res.

[12] Artanian V, Ross HJ, Rac VE, O'Sullivan M, Brahmbhatt DH, Seto E (2020). Impact of remote titration combined with telemonitoring on the optimization of guideline-directed medical therapy for patients with heart failure: internal pilot of a randomized controlled trial. JMIR Cardio.

[13] Greenhalgh T, Wherton J, Papoutsi C, Lynch J, Hughes G, A'Court C, Hinder S, Fahy N, Procter R, Shaw S (2017). Beyond adoption: a new framework for theorizing and evaluating nonadoption, abandonment, and challenges to the scale-up, spread, and sustainability of health and care technologies. J Med Internet Res.

[14] Greenhalgh T, Maylor H, Shaw S, Wherton J, Papoutsi C, Betton V, Nelissen N, Gremyr A, Rushforth A, Koshkouei M, Taylor J (2020). The NASSS-CAT tools for understanding, guiding, monitoring, and researching technology implementation projects in health and social care: protocol for an evaluation study in real-world settings. JMIR Res Protoc.

[15] (2022). Indigenous population continues to grow and is much younger than the non-indigenous population, although the pace of growth has slowed. Statistics Canada, Government of Canada.

[16] (2011). Ethnic diversity and immigration. Statistics Canada.

[17] Demography: future trends. The King's Fund.

[18] Litchfield I, Shukla D, Greenfield S (2021). Impact of COVID-19 on the digital divide: a rapid review. BMJ Open.

[19] Eruchalu CN, Pichardo MS, Bharadwaj M, Rodriguez CB, Rodriguez JA, Bergmark RW, Bates DW, Ortega G (2021). The expanding digital divide: digital health access inequities during the COVID-19 pandemic in New York city. J Urban Health.

[20] Pham Q, El-Dassouki N, Lohani R, Jebanesan A, Young K (2022). The future of virtual care for older ethnic adults beyond the COVID-19 pandemic. J Med Internet Res.

[21] Jones J, Hayes J (1999). A comparison of electronic-reliability prediction models. IEEE Trans Rel.

[22] (2021). Statistical digest of rural England: broadband. Department for Environment Food & Rural Affairs.

[23] (2021). Waiting to connect: the expert panel on high-throughput networks and remote communities in Canada. Council of Canadian Academies.

[24] Chronic heart failure in adults: diagnosis and management. National Institute for Health and Care Excellence (NICE).

[25] Afzal C, Al-Ubaydli M, Simon A, Guy B, Paul C, Kay F, Vanesther H, Eleonora H, Rosemary K, Kirk A, Lucchi G, Marriot T, Mesko B, Miles N, Nelson S, Papadakis M, Power R, Rahman R, Ratcliff F, Rose J, Stone D, Taylor C, Wilkinson T, Young N (2020). An innovator's guide to the NHS: navigating the barriers to digital health. Boehringer Ingelheim.

[26] Greenhalgh T, Fahy N, Shaw S (2018). The Bright Elusive Butterfly of value in health technology development comment on "providing value to new health technology: the early contribution of entrepreneurs, investors, and regulatory agencies". Int J Health Policy Manag.

[27] Unsworth H, Dillon B, Collinson L, Powell H, Salmon M, Oladapo T, Ayiku L, Shield G, Holden J, Patel N, Campbell M, Greaves F, Joshi I, Powell J, Tonnel A (2021). The NICE evidence standards framework for digital health and care technologies - developing and maintaining an innovative evidence framework with global impact. Digit Health.

[28] (2021). Canada's first digital health evaluation network. Canada's Drugs and Health Technology Agency.

[29] Evidence standards framework for digital health technologies. National Institute for Health and Care Excellence (NICE).

[30] Digital Technology Assessment Criteria (DTAC). NHS 75 England - Transformation Directorate.

[31] How to use the DTAC. NHS 75 England - Transformation Directorate.

[32] Innovation and technology payment. NHS 75 England - Accelerated Access Collaborative.

[33] What are integrated care systems?. NHS 75 - England.

[34] (2022). Pan-Canadian virtual care priorities in response to COVID-19. Health Canada.

[35] COVID-19 update regarding the provision of in-person and virtual care. College of Physicians and Surgeons of Ontario.

[36] Plan NLT, Watson P Chapter 5: digitally-enabled care will go mainstream across the NHS. NHS Long Term Plan.

[37] National general practice improvement programme. NHS 75 England.

[38] Department of Health and Social Care (2022). A plan for digital health and social care. GOV.UK.

[39] Joshi I (2021). Round 3 of the artificial intelligence in health and care award is now open. NHS 75 England - Transformation Directorate.

[40] Department of Health and Social Care (2022). Health secretary sets out ambitious tech agenda. GOV.UK.

[41] Starmer K (2022). A Tory party mired in scandal can't fix the NHS - but a labour government can. The Guardian.

[42] (2023). Regulatory approaches to telemedicine. Europe Economics.

[43] UK GDPR guidance and resources. Information Commissioner's Office.

[44] (2018). PIPEDA in brief. Office of the Privacy Commissioner of Canada.

[45] Bateman R (2023). GDPR vs PIPEDA. TermsFeed.

